# Designing a Private and Secure Personal Health Records Access Management System: A Solution Based on IOTA Distributed Ledger Technology

**DOI:** 10.3390/s23115174

**Published:** 2023-05-29

**Authors:** Serkan Akbulut, Farida Habib Semantha, Sami Azam, Iris Cathrina Abacan Pilares, Mirjam Jonkman, Kheng Cher Yeo, Bharanidharan Shanmugam

**Affiliations:** 1 Faculty of Science and Technology, Charles Darwin University, Darwin, NT 0909, Australia; 2 Energy and Resources Institute, Faculty of Science and Technology, Charles Darwin University, Darwin, NT 0909, Australia

**Keywords:** privacy, security, IOTA, patient health record, medical record, access management, IoT medical device, healthcare, IPFS, encryption

## Abstract

The privacy and security of patients’ health records have been an ongoing issue, and researchers are in a race against technology to design a system that can help stop the compromising of patient data. Many researchers have proposed solutions; however, most solutions have not incorporated potential parameters that can ensure private and secure personal health records management, which is the focus of this study. To design and develop a solution, this research thoroughly investigated existing solutions and identified potential key contexts. These include IOTA Tangle, Distributed Ledger Technology (DLT), IPFS protocols, Application Programming Interface (API), Proxy Re-encryption (PRE), and access control, which are analysed and integrated to secure patient medical records, and Internet of Things (IoT) medical devices, to develop a patient-based access management system that gives patients full control of their health records. This research developed four prototype applications to demonstrate the proposed solution: the web appointment application, the patient application, the doctor application, and the remote medical IoT device application. The results indicate that the proposed framework can improve healthcare services by providing immutable, secure, scalable, trusted, self-managed, and traceable patient health records while giving patients full control of their own medical records.

## 1. Introduction

The privacy and security of health records have been the main concerns of patients, as they do not want healthcare providers to be looking at their files when they do not need to [1]. Giving ownership and allowing full control of health records to patients has been one of the remedies to gain their trust in the system. However, this does not mean that privacy and security are ensured. Different techniques and technologies that can guarantee patient privacy and security are explored as options in designing systems to supplement existing PHR. In addition to this, the big data healthcare services hold has become a lucrative source for ransom and is becoming a worldwide issue. These issues are still at large, and researchers and experts are doing their best to come up with solutions that can tackle these issues.

As time flies by, these challenges become more complex due to rapid advancements in technology. New technologies keep emerging, and they all swiftly change the way people live and enable people to work more efficiently. This innovation is not ready to slow down just yet as more technologies that disrupt people’s way of life are starting to roll out one by one. Part of this technological revolution is the explosion of billions of devices around the world, and the internet has enabled these devices to be interconnected with one another. IoT technology has transformed the way people communicate and connect with each other. There are six main domains where IoT is used. These are home automation, smart infrastructure, security and surveillance, transportation, industrial application, and healthcare [2].

These domains are reaping the benefits of this advancement, and it has allowed them to grow and mature at a pace they never expected [3,4]. The healthcare industry, however, is adapting at a slower pace than others. Healthcare systems are complex, which makes the adaptation of new technologies more difficult [5,6], particularly in IoMT interoperability. Furthermore, it is an industry that nurtures and takes care of the lives of people, which requires technology to be fully developed and tested before it is considered as a potential addition or solution to their existing legacy systems.

IoT technologies are among the technologies that are being extensively used by many industries; they provide an array of benefits, such as cost-effectiveness, increased productivity, and improved efficiency [7]. It is not surprising that this technology has started to penetrate the healthcare sector at a rather gradual stride; it brings with it a promising progress. Healthcare monitoring, early diagnosis of medical issues, notification or alert systems for emergency services, and computer-assisted rehabilitation are some of its uses, to name a few [8]. It has effectively proven its worth in the healthcare sector as it becomes increasingly apparent how established this technology is in supporting health systems [9]. 

It has progressively gained significant traction since coronavirus (COVID-19) hit the entire world. Remote patient monitoring, real-time patient monitoring, and drug management are some of its uses that would have been very useful during this situation [2]. After realizing IoT’s benefits in healthcare, there has been an increased motivation to develop a framework to integrate it into healthcare [2,10,11]. However, this also brought many issues regarding PHR and IoMT interoperability. Managing device variety, scalability, data privacy, data interchange, hardware implementation and design, optimization problems, security difficulties, real-time processing, low power consumption, and data integration can be categorized as these concerns. 

This research will develop an access management system on a fully decentralized personal health records system using IOTA Tangle that will provide full control of medical records. The Patient Care Information System (PCIS) is primarily responsible for maintaining the client records at the healthcare provider organisation [12], including programmes that allow caregivers to keep track of individuals or groups of patients in a rapid, responsive, adaptable, and courteous manner while maximising available resources. A new patient can be registered in a healthcare facility through the patient registration process, which includes personal details collection, patient records management, and maintaining the register of every patient. Based on the patient’s needs, appropriate care provider resources, such as healthcare facilities (room/bed), are assigned by the Client-Resource Management Application [13]. This research mainly focuses on the hospital admission, patient discharge, and remote patient health data record when registering and retrieving personal data. People have seen the current situation where hospitals are challenged almost beyond their breaking point [14]. People who work at every healthcare service will fulfil their duty to care for people even beyond their limits, but, hopefully, with the aid of appropriate technology, the burden on their shoulders will somehow be lifted. 

The benefits of IoT in healthcare continue to grow as more and more devices become available. Some of its uses are still unexplored, and there will be applications brought about by the convergence of devices people never would have imagined. However, its uses are coupled with known challenges, such as unexpected risks related to big data, security [15], privacy, and storage due to the increasing number of users generating data [8]. The benefits outweigh the challenges, so the focus is on the challenges to be solved rather than finding an alternative solution to using this technology. Some of the known working solutions to this are restricting access to data and devices and giving ownership of data back to patients so they have full authority and control over their data. 

### 1.1. Aim of This Study

This paper aims to design a fully decentralized PHR using IOTA Tangle to secure patient medical records and IOT medical devices with an access management system that gives patients full control of their own medical records.

### 1.2. Related Works

Policies and guidelines have been in place for electronic health records (EHR) in a bid to protect their privacy and security [16]. According to Rezaeibagha et al.’s [17] findings, well-defined access control policies should be provided in addition to implementing the most appropriate architecture, framework, techniques, and policies to ensure the privacy and security of EHR. Despite the remarkable improvement it brings to the traditional healthcare system, such as reduction of medical errors, lowered healthcare costs, and improved healthcare quality [18], it has faced a lot of barriers and low user uptake. To boost the population’s confidence in and acceptance of this system, the ability for patients to manage their own records was introduced [19]. Improving the quality of care and safety, as well as empowering patients to have full control and authority over their health records, are some of the significant benefits of the widespread adoption of EHR [20].

However, there were some technical and non-technical barriers during the adoption of EHR. Providers fail to involve the users when designing the system, which leads to people finding it hard to trust the system [21]. Technological literacy discourages people, especially the elderly, from using PHR, and there is very little provision to support them in using the system [22]. The biggest barrier to date is privacy and security and not having measures in place in case there is a breach [23,24,25]. 

There have been attempts to address privacy, security, and other challenges in PHR, most of which are blockchain-based solutions. OmniPHR [26] proposed a distributed architecture by partitioning PHR in data blocks while being interoperable. This design can handle a growing number of nodes and requests without significantly affecting the delivery time. It does, however, lack in the evaluation of its security, privacy, and interoperability. Semantha et al. [13,27,28] conducted a systematic literature review on privacy by design and proposed a framework using distributed data storage and sharing for secure and scalable electronic health records management. Another patient-centred novel framework called Healthchain [29] was proposed. This one is compliant with HL7 Fast Healthcare Interoperability Resources (FHIR), which allows seamless transfers between systems following the same standard. There are six main components to this framework: patient-centred, uses permissioned blockchain, interoperable, utilizes mixed-block blockchain, uses smart contracts, and is Health Insurance Portability and Accountability Act (HIPAA) compliant. 

There are models proposed using blockchain-based access controls for personal health records. Thwin and Vasupongayya’s [30] proposal used the Ateniese, Fu, Green, and Hohenberger (AFGH) proxy re-encryption (PRE) technique as its mechanism for access control. It can apply fine-grained access controls and can revoke permission. Encrypted health records are stored on the cloud, making data available all the time, while related metadata are stored on private blockchain. Another study focusing on access control was proposed by Meier et al. [31]. All access management processes are carried out through blockchain. It gives access information to users, but data had to be stored outside due to large file sizes. Hussien et al. [32] also proposed a blockchain-based access control scheme to secure shared PHR using decentralized storage. Its access control scheme is based on smart contract-based, attribute-based searchable encryption, and it complements the system by using IPFS to allow sharing and storing of PHR without compromising security. 

With PHR, patients are more informed, and it may let them feel that they are more capable when they can request and make decisions together with clinicians. It allows them to be in control of their health-related activities [33]. This positive feedback from patients does lead to better health outcomes. However, the privacy of patients is not solely solved by giving patients control over their information. How PHR functions in healthcare, what purposes it serves, and what values it promotes need to be properly articulated. Technology plays a very important role in ensuring privacy policies are expressed precisely and unambiguously while being compliant with standards [34]. A comparative analysis between the existing solutions is presented in Table 1.

In this research, we assessed existing solutions to identify the key contexts and to compare the gaps of individual frameworks. To do so, we established a comparative analysis to highlight the inadequacies of the selected frameworks, and we identified the key contexts. The key contexts are IOTA Tangle, Distributed Ledger Technology (DLT), IPFS protocol, Application Programming Interface (API), Proxy Re-encryption (PRE), and access control. Table 1 presents a comparison of our proposed framework to the existing solutions. The key contexts of designing a private and secure personal health records access management system are derived by assessing the relevant studies. The existing solutions do not have at least one or more key contexts to ensure the privacy contexts, which are limitations for these solutions. As a result, the feasibility of the existing solutions is crucial for achieving the success of designing a private and secure personal health records access management system. In Table 1, black dots indicate that the contexts have been addressed. In contrast, the empty ones indicate that the component is either not addressed or implemented, there is a limitation, or there is still no information provided in the study. We incorporated all of the key contexts while developing our proposed solution based on IOTA Distributed Ledger Technology. The identified key contexts are as follows:

- IOTA Tangle
- Distributed Ledger Technology (DLT)
- IPFS protocols
- Application Programming Interface (API)
- Proxy Re-encryption (PRE)
- Access Control

### 1.3. Comparison with Blockchain

The majority of decentralized cryptocurrencies, including all of the more well-known ones, such as Bitcoin, Ethereum, and numerous others, demand that anybody conducting a transaction on the network pay a charge for the services offered. The explanation is that a miner charges a fee for each transaction in the blockchain as proof of their effort. The role of the miner is to validate the transactions of users by computing specific algorithms and to produce blocks of blockchain. As the number of users grows, so will the fee. IOTA, however, eliminates miners and allows users to confirm each other’s transactions with a small amount of proof of work, which enables feeless transactions. IOTA defines its principle as “Help others, and others will help you; however, if you choose not to help others, others will not help you either” [45].

Both blockchain and IOTA employ Distributed Ledger Technology; however, the way they use DLT is significantly different from one another. IOTA adopts a DAG structure, whereas blockchain uses a chain type of block. Thus, blockchain has speed, scalability, block size, interoperability, and sustainability restrictions, but IOTA overcomes those issues by using the DAG structure [46]. While just one block is utilized for transaction recording in the blockchain, DAGs allow for the simultaneous existence of several nodes.

Blockchain offers promising potential solutions, but it also has challenges in terms of cost, scalability, and flexibility in data access management. IOTA and the Tangle have characteristics that can overcome some of the challenges or limitations of blockchain. Exploring this technology as a solution also comes in handy with the evolving IoT devices that are intended for the healthcare industry, such as emergency sensors, remote patient monitoring devices, and health and fitness wearables, to name a few [47].

## 2. Materials and Methods

This section will describe the technologies used in the proposed framework, using IOTA as its key element. In this study, IOTA Distributed Ledgers are used to develop a patient-based access management system. IoT devices will be considered in designing the framework, as these patient-data-generating devices may be used at any point throughout the patient’s journey.

### 2.1. Comparison with Blockchain

#### 2.1.1. IOTA Tangle

**Figure 1:**
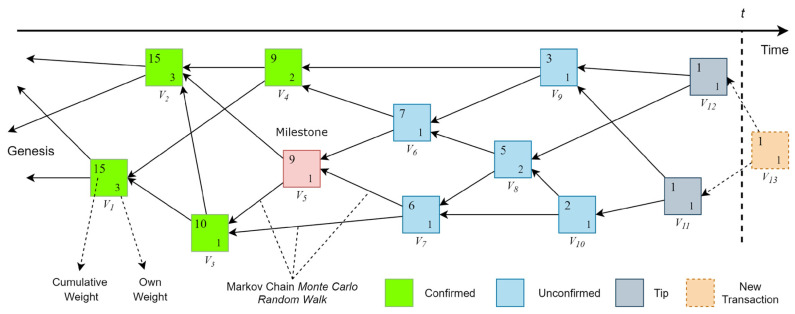
IOTA Tangle.

IOTA Tangle consists of tips, confirmed, unconfirmed, coordinator, and milestones nodes [48], as shown in Figure 1.

In the tangle, tips are unconfirmed new transactions. Whenever a new transaction is created, the node selects two other transactions using the Markov chain Monte Carlo (MCMC) Random Walk algorithm. The algorithm traverses the tangle and chooses the most weighted nodes to eliminate lazy tips. The node confirms that chosen transactions are not conflicting, and then a cryptographic puzzle, which is a finding nonce, needs to be solved to join the node tangle. After the node has joined the tangle, it becomes a new tip. Every node in the tangle has its own cumulative weight [45]. For instance, the cumulative weight of V6 can be calculated as Equation (1).

V6(cumulative weight) = V6(own weight) + V8 + V9 + V10 + V11 + V12 V6(cumulative weight) = 1+ 2 + 1 + 1 + 1 + 1 = 7(1)

A higher number of weights shows the importance of the node in the tangle. Milestones are checkpoints created by coordinators to validate transactions. Therefore, transactions must be validated either directly by coordinators or indirectly by milestones [48]. Currently, IOTA is using coordinators, and it is issued by the IOTA Foundation. For that reason, IOTA cannot be considered fully decentralized. However, the IOTA Foundation introduced Coordicide, an algorithm to eliminate coordinators and make the tangle fully decentralized [49].

#### 2.1.2. IOTA Address Generation

**Figure 2:**
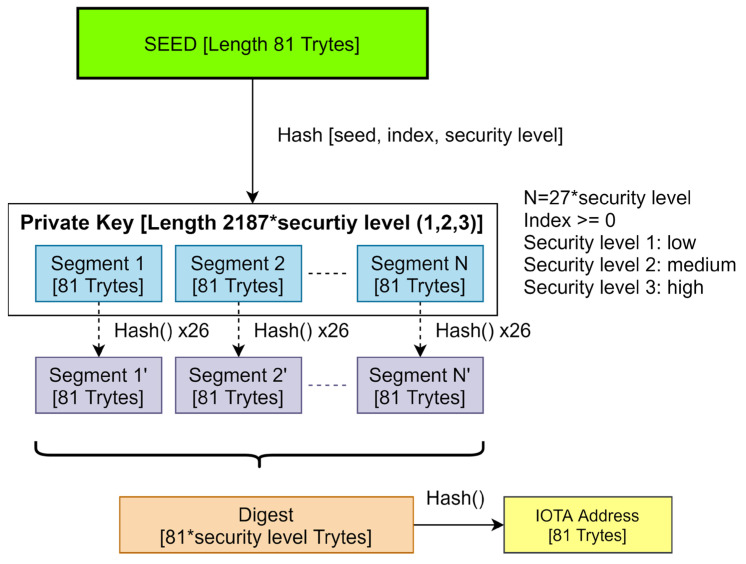
IOTA address generation.

In Figure 2, IOTA addresses are created from a seed, and a seed is generated by patients with random seed generators. 

A seed is the patient’s private key for IOTA transactions; hence, it must be produced and maintained safely. The length of a seed is 81 trytes. A tryte is 3 trits (−1, 0, 1); thus, there are 3^3^ possible outcomes. Therefore, the Tryte Alphabet consists of 27 characters (9ABCDEFGHIJKLMNOPQRSTUVWXYZ) [29]. There are 27^81^ possible seeds that can be generated. In comparison to Bitcoin’s seed generation (2^256^), IOTA offers a wider range of keys to enhance each key’s uniqueness (Equation (2)).

3^243^(8.718964e + 115) > 2^256^ (1.1579209e + 77) (2)

For address generation, IOTA uses quantum computer proof Winternitz One-Time Signature, which is a hash-based algorithm [50,51]. Figure 2 demonstrates private key generation with the given index number, security level, and seed. First, using the seed (private key) with an index number, sub-seeds are created by hashing them. Sub-seeds are hashed again, and N numbers of sub-private keys are created. After the private key is broken down to N segments, it is hashed 26 times with the World of Tanks (WOT) algorithm and digested with the Keccak-384 based Kerl hash algorithm [52]. After final hashing, an 81-tryte-length public key (IOTA Address) is created. Due to the nature of One-Time Signatures, when a transaction is digitally signed, a part of the sender’s private key is revealed to the receiver. Therefore, addresses that are used for spending should not be used again [53]. However, an IOTA address can receive many transactions without revealing private keys. 

After IOTA Addresses are created, patients can then use one of the IOT addresses for health records.

#### 2.1.3. IOTA Message Frame

**Figure 3:**
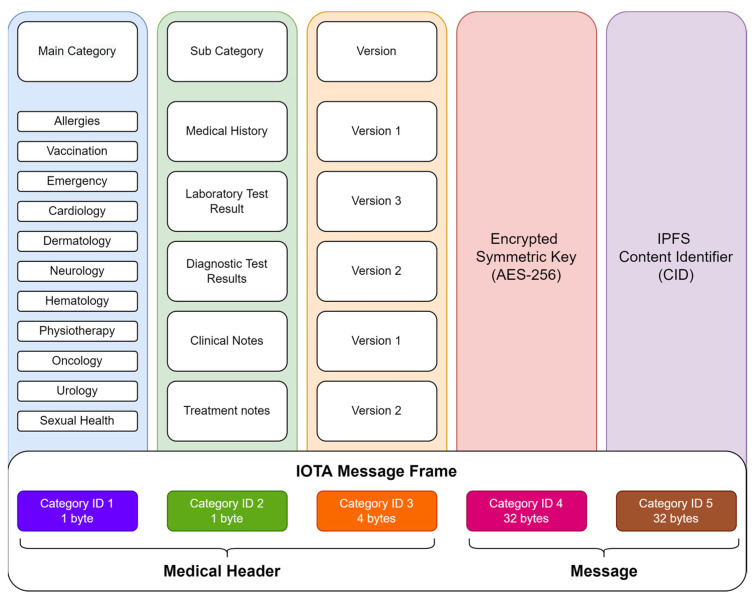
Medical header recorded in IOTA transaction message.

In the proposed framework, each new patient record is encrypted with a new Advanced Encryption Standard (AES)-256 symmetric key. Encrypted files are stored in IPFS together with a corresponding content identifier (CID) collected. Then, the medical header is created by categorizing new data into three sections, such as main category, subcategory, and version number, as shown in Figure 3. After the medical header is created, it is combined with IPFS CID and encrypted through AES-256 symmetric key. Using a universally unique identifier (UUID), the header goes through another encryption. The encrypted medical header is recorded in an IOTA message and saved as a transaction in a patent IOTA address.

#### 2.1.4. IOTA Masked Authenticated Messaging (MAM)

**Figure 4:**
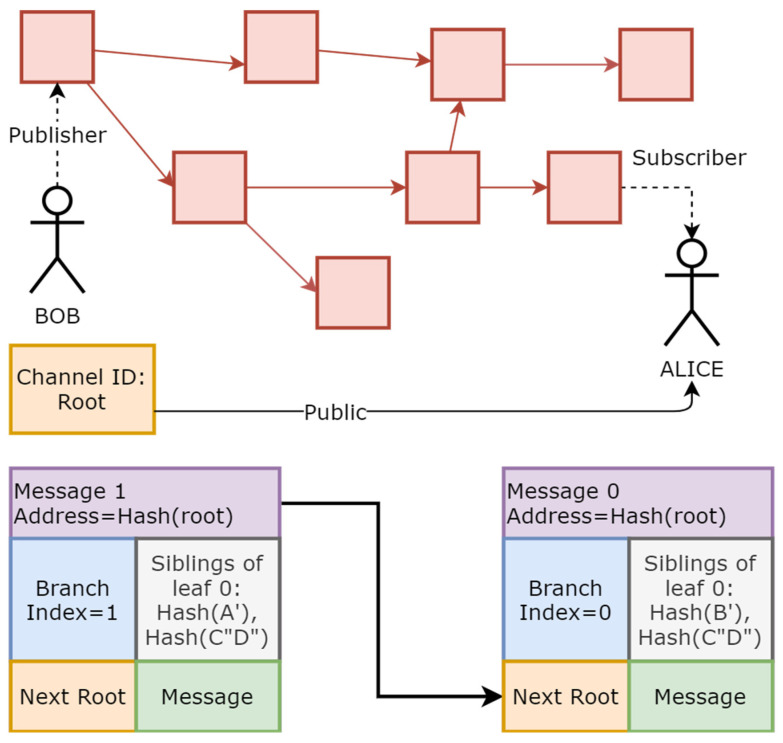
IOTA MAM messages and Gossip Protocol.

IOTA MAM is developed on the IOTA main network as a second layer to share data across the network [47]. Anyone in the network can create a channel, and MAM messages through the network are based on Gossip Protocol [54], as shown in Figure 4. IOTA MAM provides three different types of communication channels: public, private, and restricted. In public mode, a root is shared publicly, and messages are encrypted with a root. Therefore, anyone with a root address can access the channel and read the message. In private mode, the root address is hashed; thus, everyone can listen, but only subscribers with the root key can decrypt the messages. In restricted mode, the root is hashed, and messages are encrypted with a side key [47]. An example of a data stream is illustrated in Figure 4. Each message contains a hashed next root (private or restricted Mode).

#### 2.1.5. Proof of Work

It is mentioned that IOTA bundles consist of input and output transactions, and each transaction is signed with a private key. After this, using weighted random work (MCMC) [38], two tips are selected where they are leaf nodes of the confirmed transaction. Then, confirmed transactions that are found during the random work are assigned as branch and truck transactions to later calculate the nonce. IOTA uses the Curl algorithm for Proof of Work (PoW) to calculate nonce. The purpose of this PoW algorithm is to avoid spam and Sybil attacks [48].

#### 2.1.6. Smart Contracts

Smart contracts are software codes that automatically execute when certain conditions specified by the developer are met [55]. Users can operate a permissioned smart contract chain that is validated by a committee in IOTA smart contracts. Nodes in the committee can be selected, or users can use their own committees to run smart contracts. In the proposed framework, smart contracts can be validated by nodes of committees planted in hospitals. There are three smart contracts. The first smart contract is created by hospitals to charge patients for hospital expenses. The second smart contract, which is linked to smart contract 1, is created by the Patient Data Visualizer (PDV) to assign IoT devices to patients. Every IoT device used during a hospital stay is charged separately to the patient. The third smart contract created by a patient allows authorized entities to access patient health records.

#### 2.1.7. Private–Public Key Management

In the framework, the Proxy Re-encryption (PRE) method is proposed for key management. Proxy Re-encryption is a method whereas proxy server converts cyphertext A (C_A_), which is encrypted with $pk_{A}$, to cyphertext B (C_B_), which can be decrypted with $sk_{B}$ using a re-encryption key ($rk_{A\to B}$) [44,56]. Proxy only requires cyphertext A and the encryption key, which is created with $sk_{A}$ and $pk_{B}$ outside of the proxy. Therefore, the owner of cyphertext A can share secret data without revealing the private key or secret data. The key concept is to disclose the least data possible to proxy, because it is an untrusted platform, and to allow it to execute a key change from $sk_{A}$ to $sk_{B}$ to decrypt cyphertext A. The algorithm below explains the Proxy Re-encryption algorithm, which can be used in the framework [57,58].

- Key Generation:

Let $G_{1}=\langle g\rangle$ a cyclic group of prime order q.

Patient private key $sk_{a}=a\;\in \;Z_{q}^{*}$ randomly selected and public key $pk_{a}=g^{a}$

Doctor private key $sk_{b}=b\;\in \;Z_{q}^{*}$ randomly selected and public key $pk_{b}=g^{b}$

$r\;\in \;Z_{q}^{*}$ randomly selected. Z=e(g,g)

(3) $$rk_{A\to B}={\left(g^{b}\right)}^{1/a}=g^{b/a}\;\in \;Z_{q}^{*}$$

- Encryption:

(4) $$\mathrm{Let}\;m\;\in \;G_{2}.\;\mathrm{Encrypted}\;\mathrm{text}\;C_{a}=\left(Z^{r}.m,g^{ra}\right).$$

- Decryption (Patient):

(5) $$m=\frac{Z^{r}.m}{e(g^{ra},g^{1/a})}=\frac{Z^{r}.m}{Z^{r}}$$

- Re-encryption:

(6) $$\begin{array}{l} C_{a}\;\to Proxy\;Server\to \;C_{b}\\ \left(Z^{r}.m,g^{ra}\right)\to \;\left(Z^{r}.m,e\left(g^{ra},rk_{A\to B}\right)\right).\\ C_{b}=(Z^{r}.m,e\left(g^{ra},g^{b/a}\right))\\ C_{b}=(Z^{r}.m,Z^{rb}) \end{array}$$

- Decryption (Doctor):

(7) $$\;\;m=\frac{Z^{r}.m}{{\left(Z^{rb}\right)}^{1/b}}\;$$

**Figure 5:**
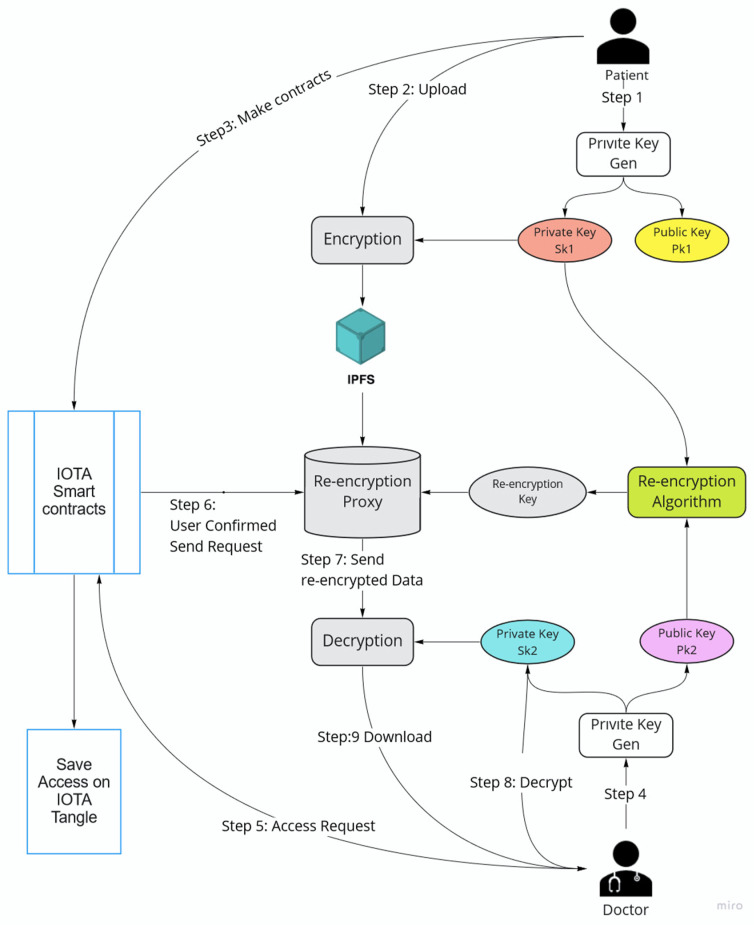
Doctor–patient key exchange with Proxy Re-encryption.

In Figure 5, the key exchange with the doctor is demonstrated. For instance, first the patient creates ($sk_{a}$, $pk_{a}$) private and public key pairs. Using $pk_{a}$ public key, the patient encrypts a symmetric key for patient health record encryption before storing the record in IPFS. Then, the patient creates a re-encryption key ($rk_{A\to B}$) using $sk_{a}$ and the doctor’s public key ($pk_{b}$). After that, cyphertext A and $rk_{A\to B}$ are stored in IOTA smart contracts. If the doctor’s access request is confirmed, the smart contract sends cyphertext A and $rk_{A\to B}$ to proxy to convert cyphertext A to cyphertext B. Finally, the doctor can decipher ciphertext B using their private key ($sk_{b}$).

#### 2.1.8. IPFS and File Management

In current websites and computers, the location-based addressing method is used to access content [40,59]. For instance, to access a website, the client enters the Uniform Resource Locator (URL) to the browser. The URL provides the hostname and specific location, which is a directory, and then it points to a file. This type of network is called client-server communication, and it is a star network topology where clients are connected to a centralized server [40]. Data stored in centralized storage may not be available if the content provider deletes the content or if the data might have been manipulated by hackers. Thus, centralized storage systems become undesirable due to a single point of failure.

**Figure 6:**
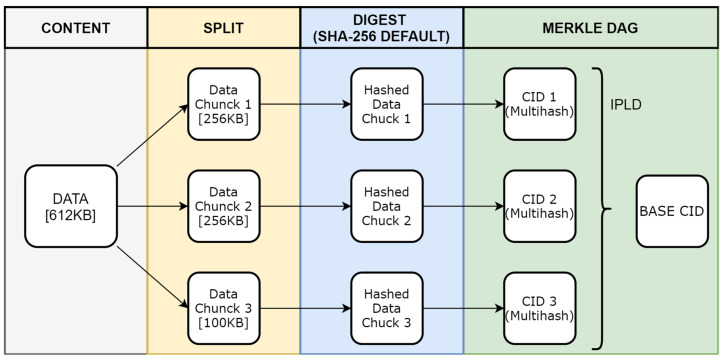
IPFS content identifier.

IPFS is a technology that is currently used for content addressing to access data [41]. It eliminates a single point of failure and uses the Merkle Tree algorithm [60] to ensure data integrity. It also uses peer-to-peer (P2P) network architecture to distribute pieces of the content over the network. The smallest piece in IPFS is 256KB. IPFS uses CID to address those small pieces. CIDs are created by hashing algorithms, as shown in Figure 6. Using the InterPlanetary Linked Data (IPLD) model, multiple CIDs of divided small data are linked to one CID. This final CID can be used to access the data stored in a distributed network. 

**Figure 7:**
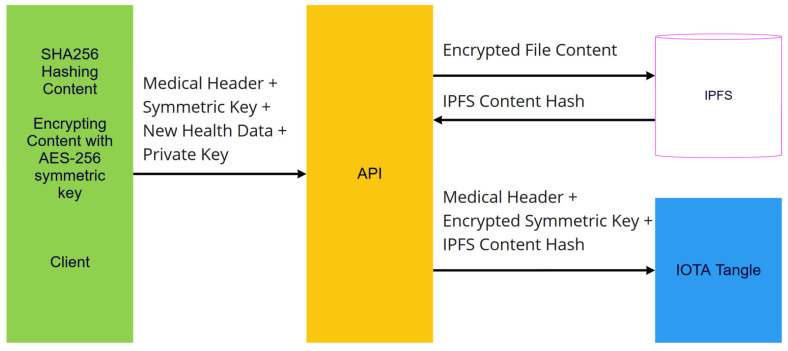
IOTA and IPFS data post diagram.

**Figure 8:**
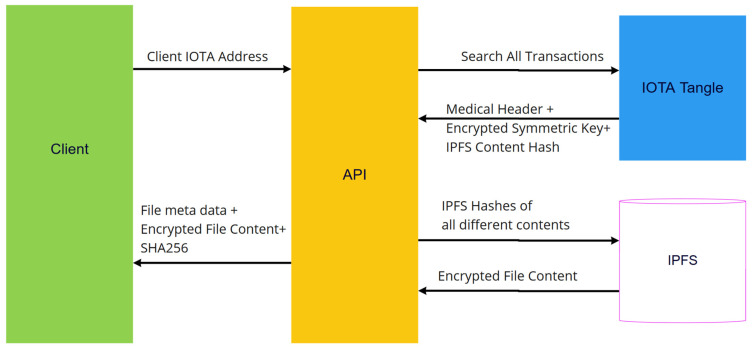
IOTA and IPFS data retrieval diagram.

To store medical data in IPFS, there is an Application Programming Interface (API) that has to be developed. This API manages new health data encryption using a client public key, medical header creation, hashing content, storing encrypted new health data in IPFS, and storing encrypted IOTA message frames in the IOTA Tangle, as displayed in Figure 7. It is also responsible for retrieving the IOTA message frame from IOTA and encrypting this frame to retrieve stored data from the IPFS server, as is shown in Figure 8.

### 2.2. Proposed Framework for Patient Health Records Access Management System

**Figure 9:**
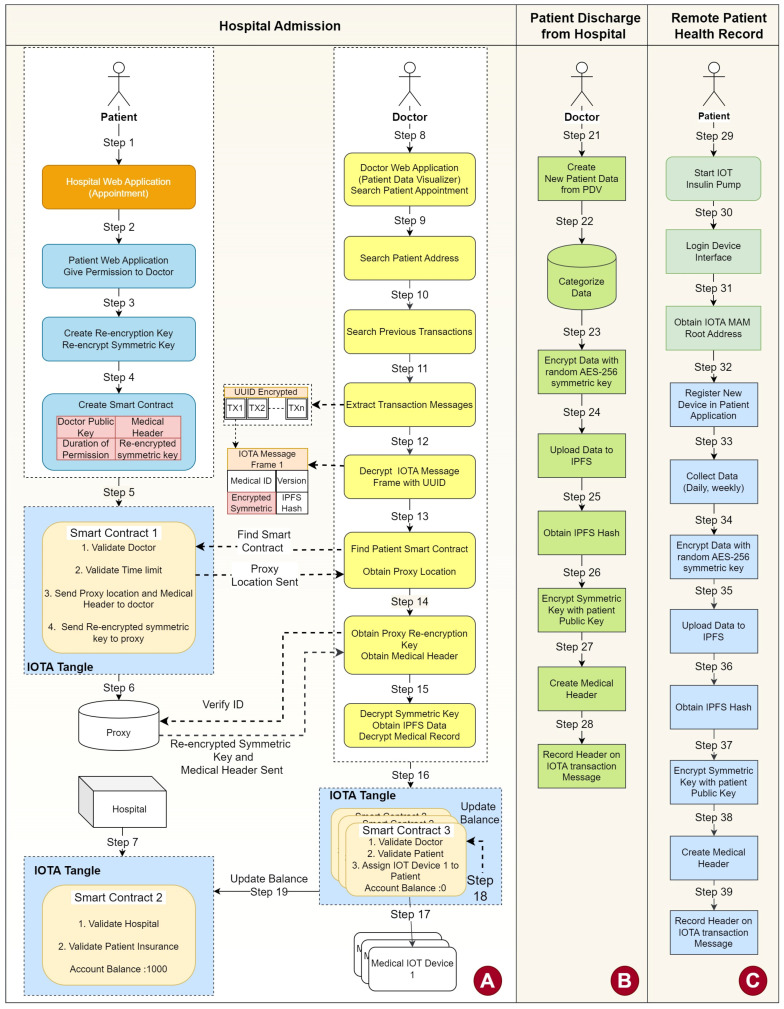
Proposed framework.

The proposed framework consists of three workflows: Hospital Admission, Patient Discharge, and Remote Patient Health Data Record. The architecture and deployment of the system are illustrated in Figure 9.

### 2.3. Actors and Main Objects of the Framework

#### 2.3.1. Patient

The patient interacts with a web application and creates smart contracts for chosen health data to grant authorization to a specific user. There are two types of authorization that a patient can grant. The first is access only, while the second is access and post new data. This approach differs from current systems, where authorized users can independently post new data without the consent of the patient.

#### 2.3.2. Doctor

The doctor is an authorized person who needs to access specific patient data. Using a PDV device, they can send a request to a patient for permission to access their data.

#### 2.3.3. Hospital

Hospitals create smart contracts to charge patients for specific services provided during their appointment.

#### 2.3.4. Web Appointment

This is used by patients to book an appointment with a doctor. The assigned doctor will receive a public key after an appointment has been approved. Hospitals also create smart contracts with the information provided by patients, such as IOTA addresses.

#### 2.3.5. QR Scanner

This is an IoT device available at the hospital where the patient is admitted. The device scans the patient’s IOTA address and then the patient selects, or the device assigns, the doctor to the patient (if not already done so via web appointment). The doctor’s public key can also be exchanged with this device. Furthermore, it investigates previous transactions made with a patient’s IOTA address to extract previous health records.

#### 2.3.6. Smart Contracts

The framework introduces three distinct smart contracts for verifying patients and authorizing people (doctors), monitoring medical IoT device usage, billing patients for hospitalization, and patient–doctor key exchange using the PRE algorithm. 

#### 2.3.7. Patient Data Visualizer (PDV)

This device as shown in section A of Figure 9; it analyzes the medical header and categorizes medical data collected from the IPFS server. For instance, it shows patient test results under corresponding main categories (hematology, allergies, vaccination, etc.) to doctors, as illustrated in Figure 3. Doctors can then make a diagnosis or issue new tests.

#### 2.3.8. IPFS

Different sorts of encrypted patient data are stored independently in IPFS servers.

### 2.4. Description of Workflows Used in the Proposed Framework

#### 2.4.1. Hospital Admission

**Figure 10:**
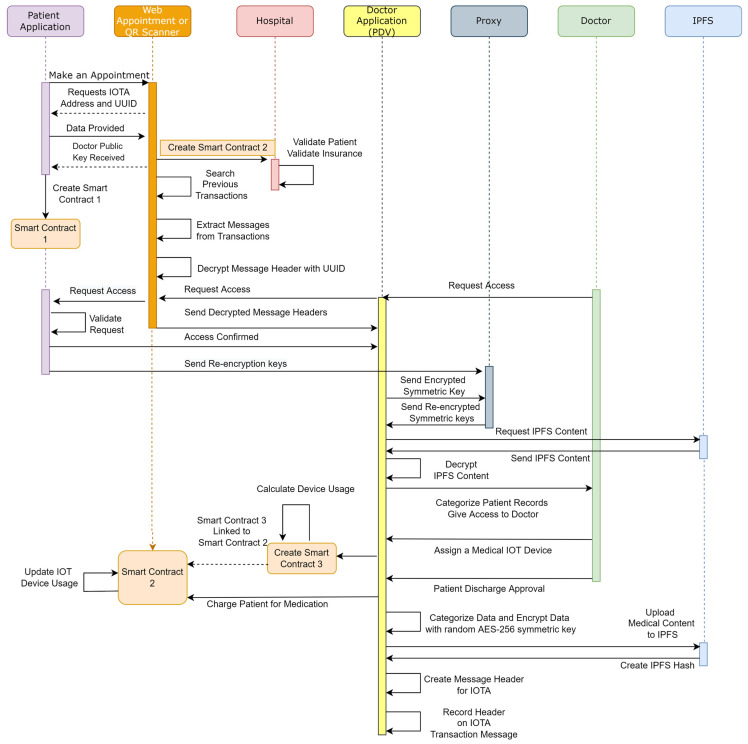
Sequence diagram of objects used in hospital admission.

The steps of patient admission are shown in the Hospital Admission Framework, which is section A of Figure 9. The interaction of objects is demonstrated in Figure 10, and all of the steps involved in the workflow are listed in Table 2. 

Patients must first provide an IOTA address that is specifically designated for patient health records in the provided framework. Section A of Figure 9 shows that there are two ways to accomplish this. The first is to use an online appointment application before coming to the hospital or to scan a QR code generated by a patient mobile application using a QR Scanner device while at the hospital. In both approaches, the patient provides an IOTA address and UUID, and the hospital delivers doctor information and the public key.

**Figure 11:**
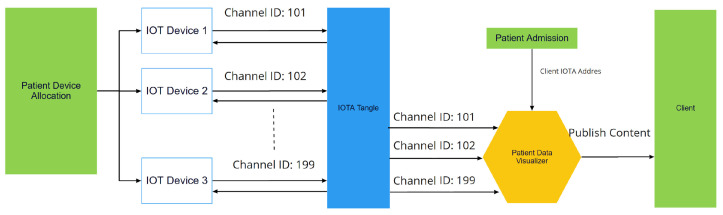
IoT medical device communication with patient data visualizer using IOTA MAM.

After a patient has been admitted to the hospital, every IoT device used in the process is connected to PDV using the IOTA MAM Protocol. PDV is responsible for recording every test result and diagnosis that comes from IoT devices. It can be seen in Figure 10 and Figure 11 that when a patient is discharged, these data will be categorized, encrypted, and stored in IPFS.

#### 2.4.2. Patient Discharge

**Figure 12:**
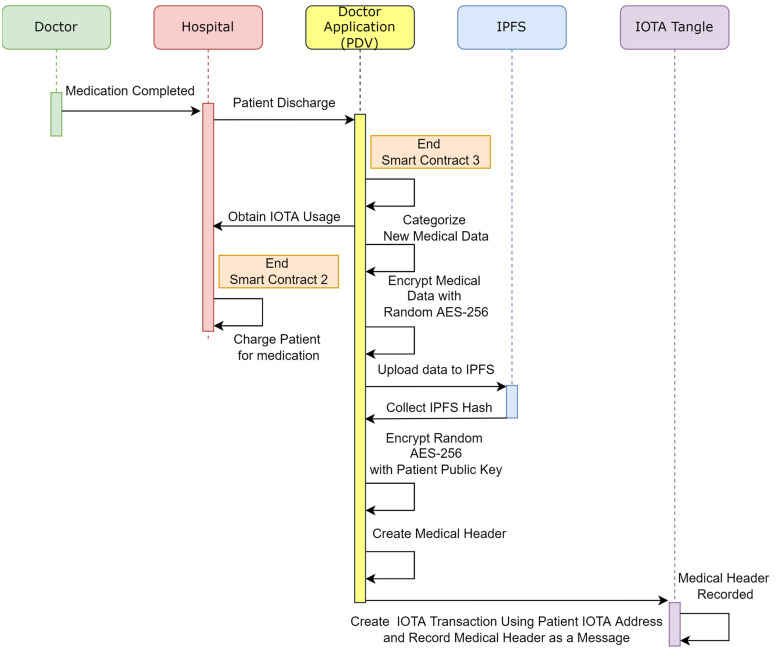
Sequence diagram of objects used in patient discharge.

Section B of Figure 9 and Figure 12 illustrates the process, and Table 3 lists the steps for this workflow. Once a patient has completed medical treatments, the doctor discharges the patient through PDV. Then, PDV informs Smart Contract 2 of the patient discharge. Smart Contract 1 is linked to Smart Contract 2 to finalize the patient balance and to make the final transaction for the cost of treatment. 

At the same time, PDV examines whether any health data have not yet been published to IPFS. If this is the case, first, it will categorize the data as instructed in section IOTA Message Frame, and then categorized data will be encrypted with the AES-256 symmetric key. It will then publish encrypted data to IPFS. Using a patient public key, it encrypts AES-256 symmetric keys. After that, the medical header, encrypted symmetric key, and IPFS hash are combined and encrypted with patient UUID. Then, it stores the encrypted IOTA Message Header in IOTA tangle using a patient IOTA address. In this process, PVD uses API, as explained in section IPFS and File Management in Figure 7.

#### 2.4.3. Remote Patient Health Data Record

**Figure 13:**
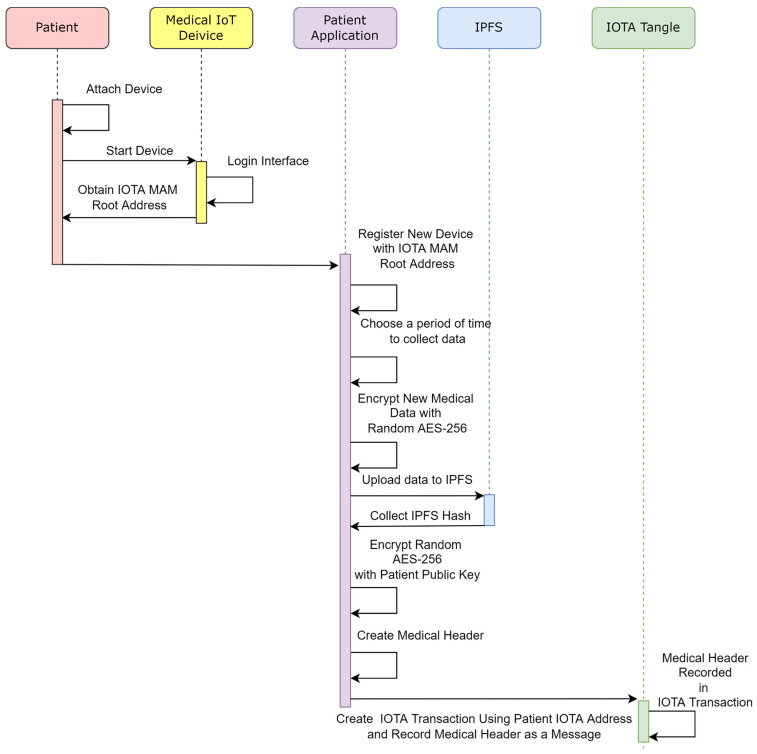
Sequence diagram of objects used in Remote Patient Health Record.

There might be patients who need to be monitored remotely. Therefore, IoT devices must be compatible with IOTA MAM. For instance, in the proposed framework shown in section C of Figure 9, patients can connect IoT devices to mobile applications and receive data from devices at a set period of time. After data are collected, data can then be stored in IPFS. Figure 13 demonstrates how a patient can remotely store IoT data to IPFS, and the steps are enumerated in Table 4.

## 3. Results

In this section, the prototype applications are demonstrated, and the results are presented. There are four applications in total to simulate the framework. The first application is the Hospital Web Application, where patients can schedule an appointment with a doctor on a specific day and time. Patients have to provide a UUID and Patient IOTA Address to the application. The Hospital Web Application shares this information with the Doctor Application, which is the second application. Using this application, doctors can download permissioned patient records using IOTA Tangle, IPFS, and Proxy Re-encryption libraries. Doctor can also assign IoT medical devices to the patient during medication. The third application is the Patient Application for patients to manage their medical records and to grant access to selected doctors. Patients can also use this application to record their health records remotely using IoT medical devices. The fourth application is developed to simulate IoT medical devices, such as a blood glucose monitor. This application connects to an IOTA node and uses IOTA MAM technology to publish medical data for a period of time. These data can be collected from the Patient Application using the IOTA MAM root address generated in the Remote Medical IoT Device Application.

### 3.1. Application 1: Web Appointment with Hospital

#### Creating Web Appointment

**Figure 14:**
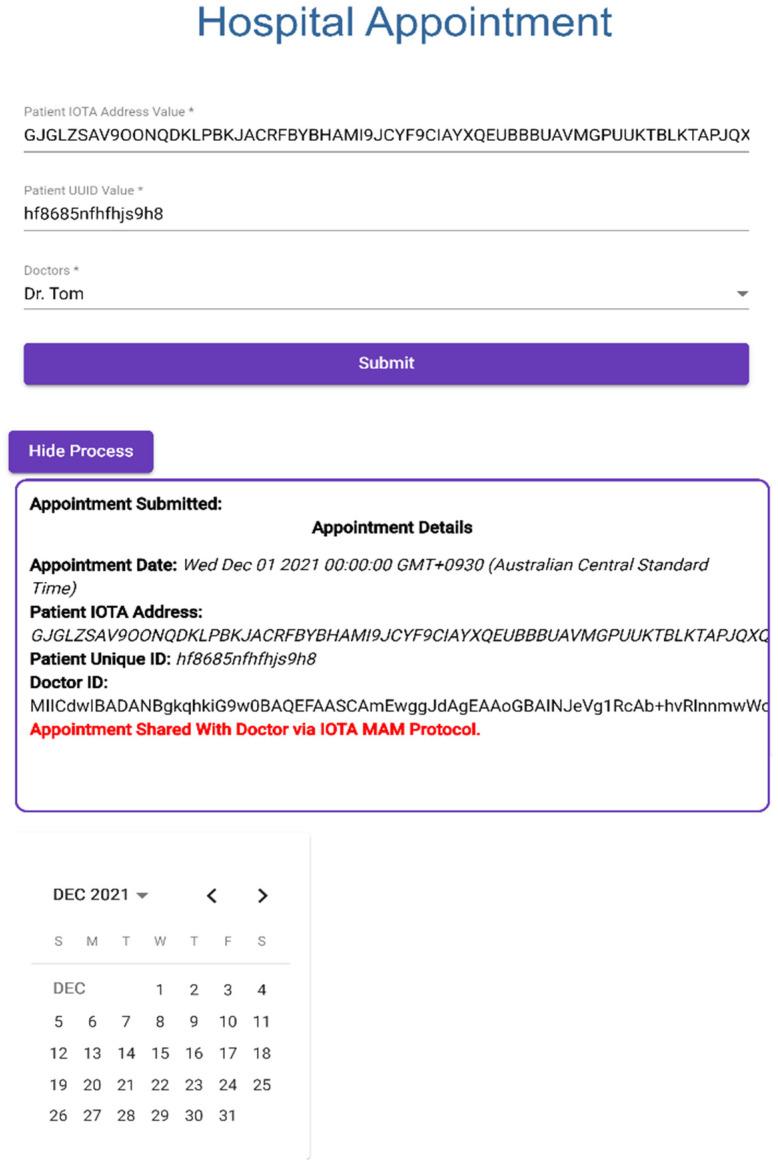
Creating Web Appointment through Hospital Application.

- Patient makes an appointment with doctor through hospital appointment system.
- Patient provides IOTA Address and UUID.
- Web appointment application creates a channel with Doctor Patient Medical Data Visualizer application via IOTA MAM protocol to submit patient information with encrypted private MAM channel; this process is shown in Figure 14.

### 3.2. Application 2: Doctor Patient Medical Data Visualizer 

This application is developed for doctors to visualize patient medical records and to assign IoT devices to a patient. 

#### 3.2.1. Creating Doctor Profile

**Figure 15:**
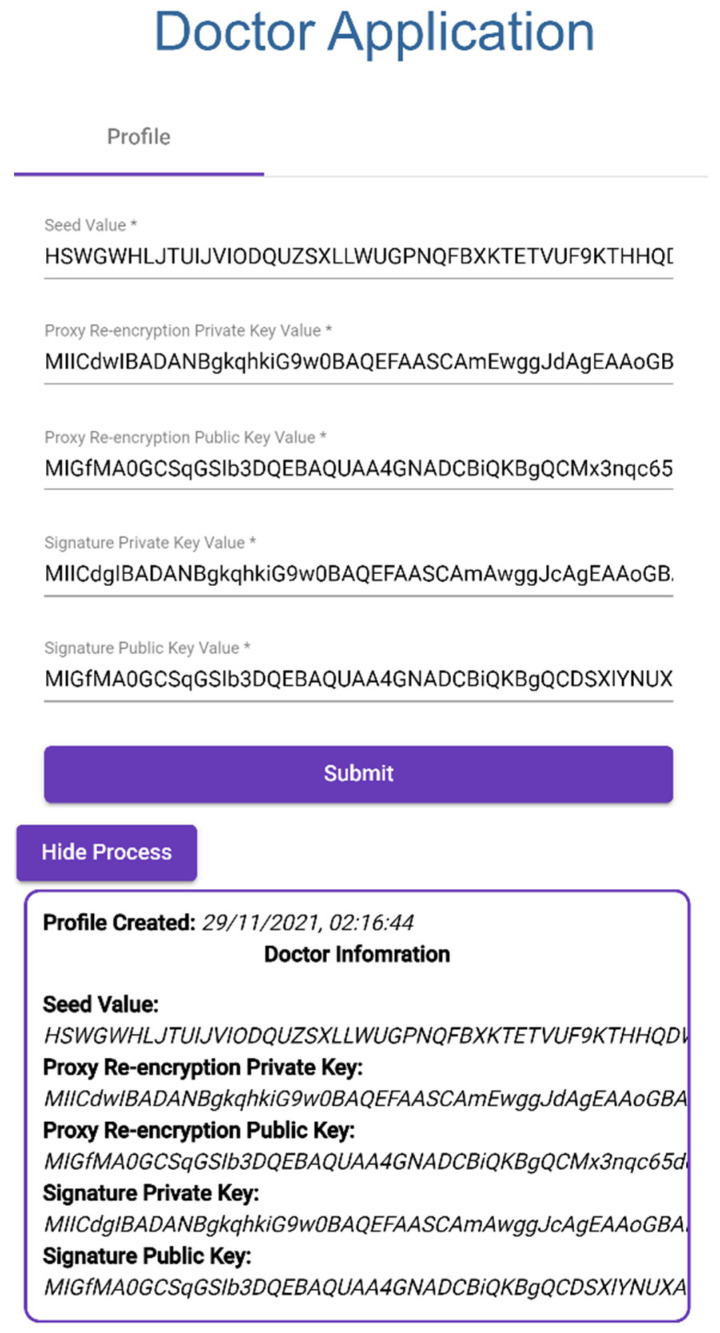
Creating Doctor Profile.

- Doctor provides IOTA Seed.
- Proxy Re-encryption Private Key.
- Proxy Re-encryption Public Key.
- Signature Private Key.
- Signature Public Key, as shown in Figure 15.

#### 3.2.2. Finding Patient Appointments and Retrieving Patient Medical Records

**Figure 16:**
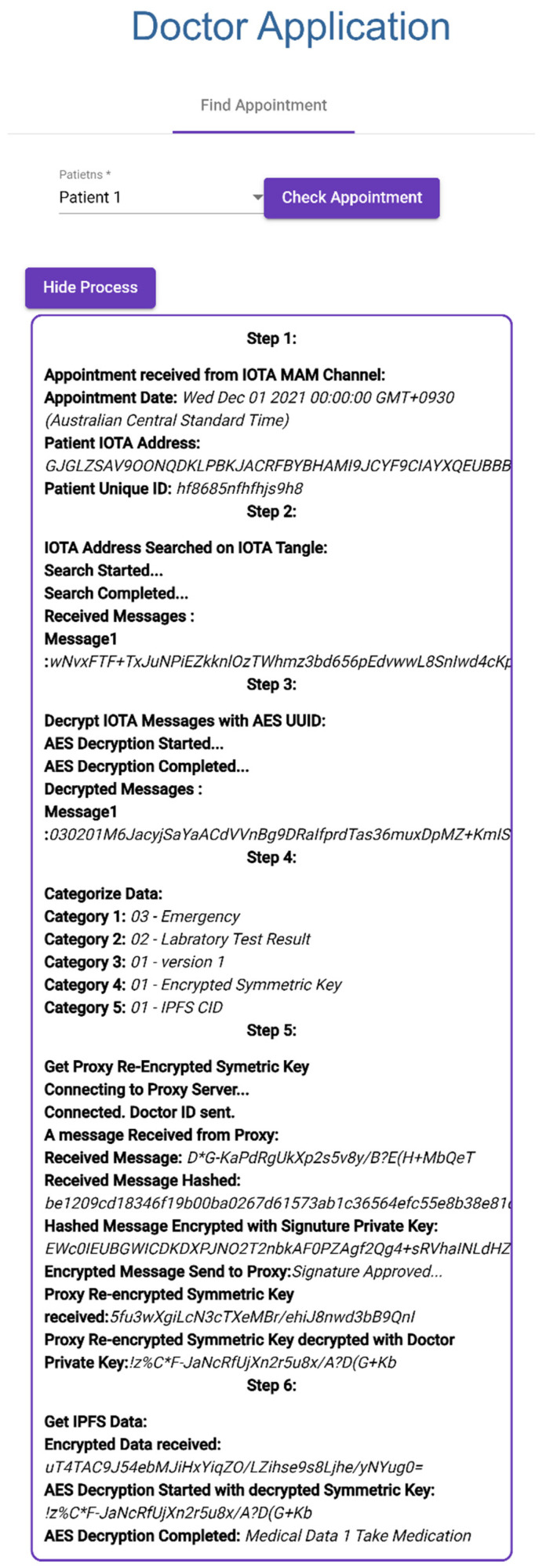
Finding Patient Appointment.

- Doctor selects patient appointment, as can be seen in Figure 16 Step 1.
- Patient IOTA address and UUID are collected from IOTA MAM Channel.
- Patient IOTA address is searched in IOTA Tangle.
- Encrypted IOTA messages are collected from past transactions (Step 2).
- Received IOTA messages are decrypted with patient UUID (Step 3).
- Decrypted IOTA messages are allocated to medical data categories (Step 4).
- Doctor application connects to proxy Re-encryption server.
- Doctor application sends Doctor ID (Doctor Signature Public Key) to server for identification.
- Proxy server sends random data to be signed by doctor.
- Message is hashed using SHA-256 by doctor application and hash signed with Doctor Signature Private Key.
- Proxy server decrypts with Doctor Signature Public Key to confirm Doctor ID.
- If Doctor ID is confirmed, proxy server sends Proxy Re-encrypted Symmetric Key.
- Proxy Re-Encrypted Symmetric Key is decrypted by Doctor Proxy Re-encryption Private Key (Step 5).
- Encrypted PHR is stored in IPFS and is collected using IPFS CID.
- Encrypted Patient Health Record is decrypted with decrypted Proxy Re-encrypted Symmetric Key (Step 6).

#### 3.2.3. Visualizing Patient Medical Record

**Figure 17:**
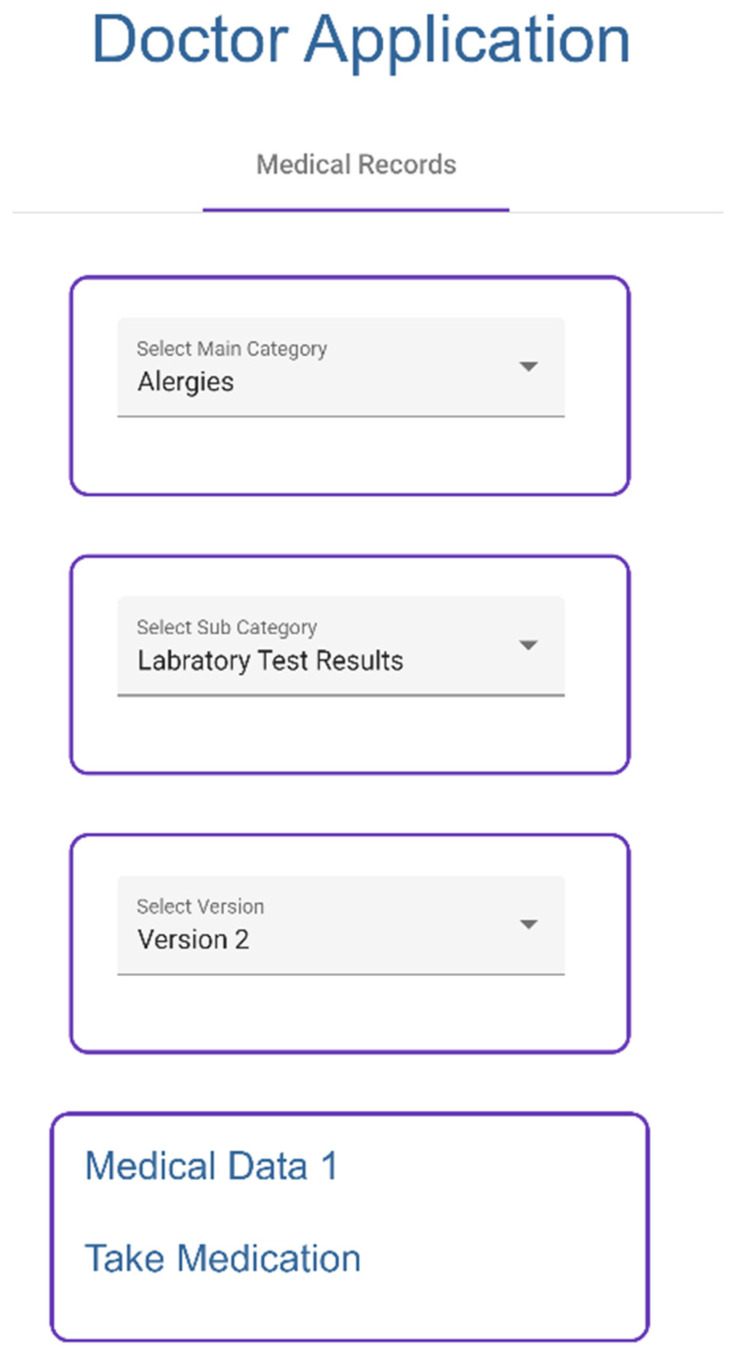
Visualizing Medical Record—Doctor Application.

- Doctor selects categories (Figure 17) to access medical records, which are collected from IPFS.

#### 3.2.4. Assigning an IoT Device to a Patient

**Figure 18:**
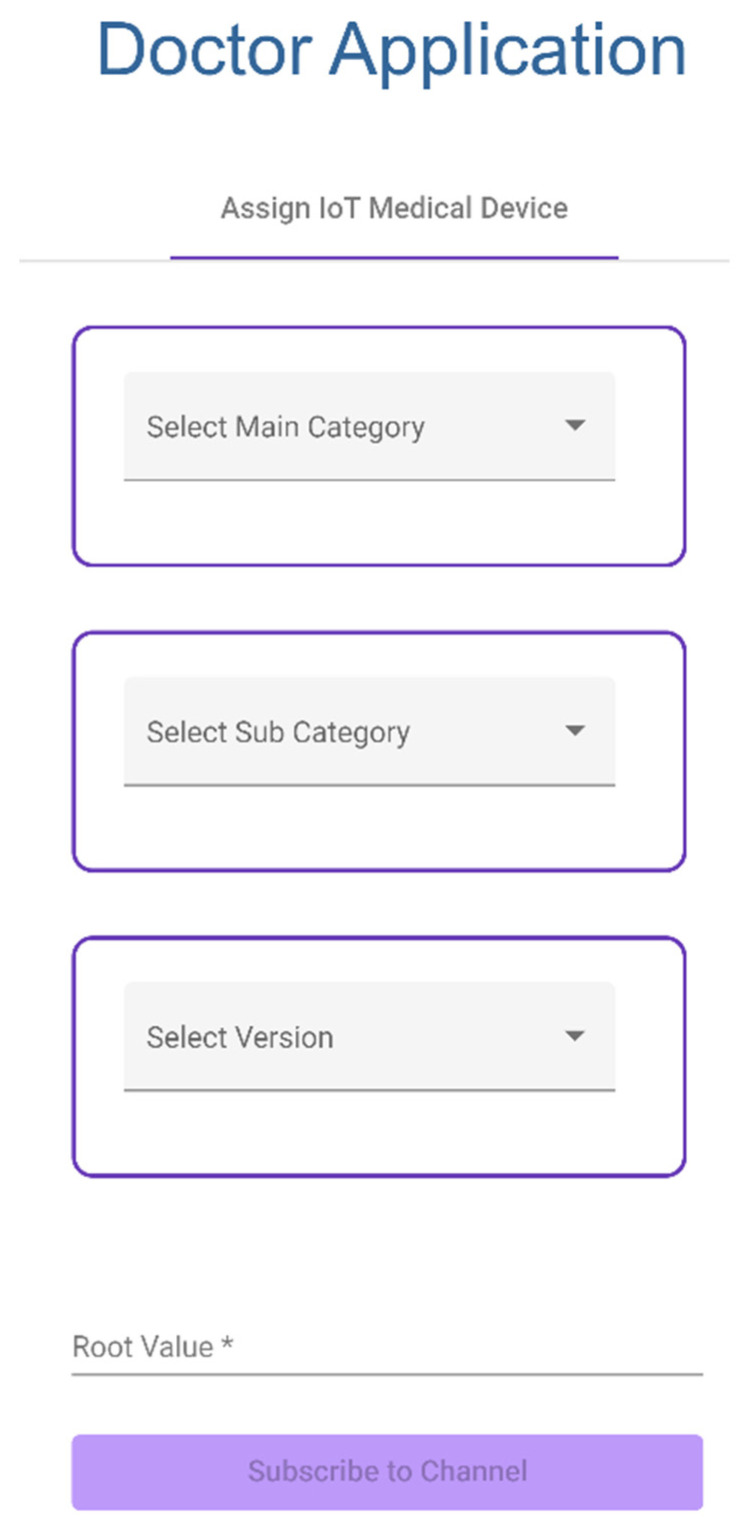
Assigning an IoT Device to a patient.

- Doctor selects a category and enters the root address of the device to assign IoT Medical Device to a patient.
- Doctor application subscribes to an IoT device channel through private IOTA MAM channel (Figure 18).

#### 3.2.5. Creating New Medical Data

**Figure 19:**
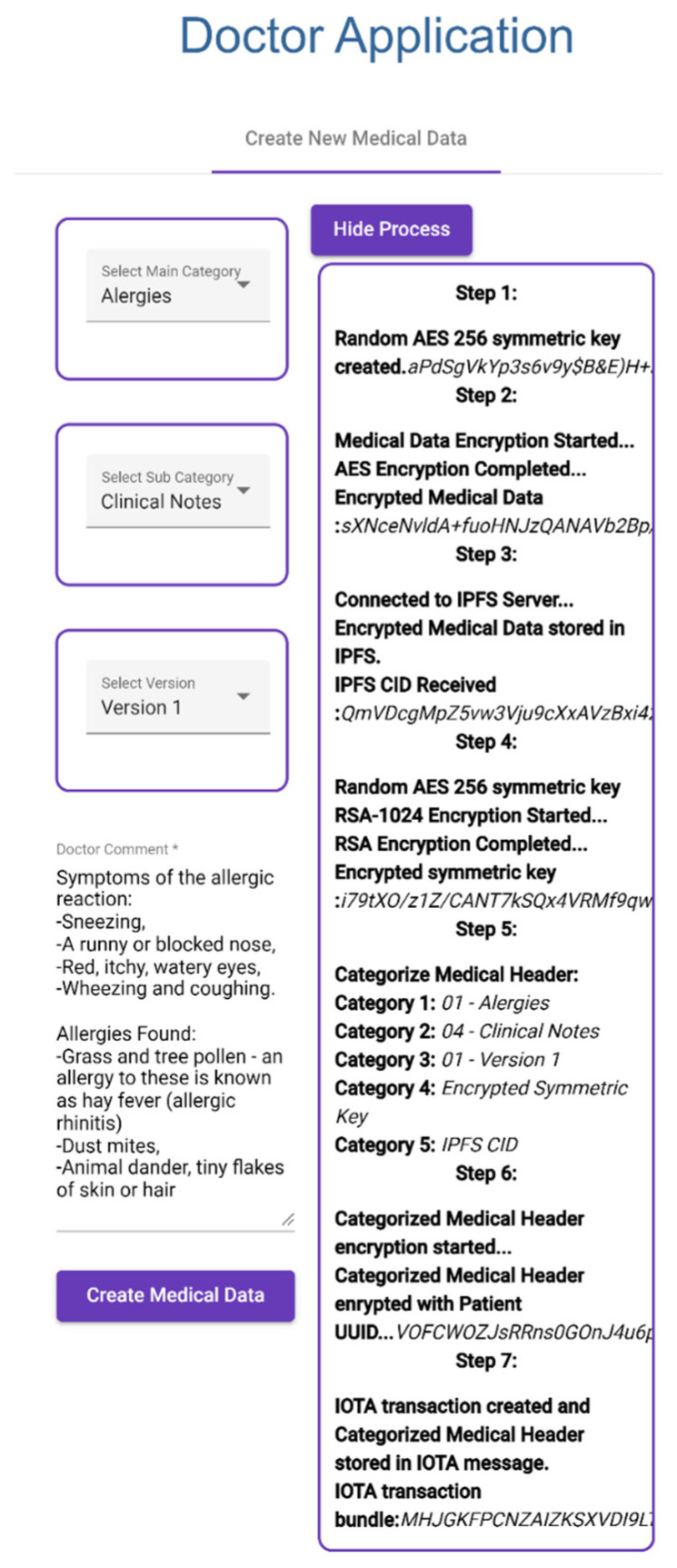
Creating New Medical Record.

- After the doctor has selected a patient from Find Appointment, the doctor selects categories for new medical data and then writes clinical notes and creates medical data, as shown in Figure 19.
- Created medical data are encrypted with random AES-256 symmetric key.
- Encrypted medical data are stored in IPFS and then IPFS CID is collected.
- Symmetric key is encrypted with patient public key.
- Selected categories, encrypted symmetric key, and IPFS CID are recorded in patient IOT address as a transaction message.

### 3.3. Application 3: Patient Medical Record Access Control

This application is developed for patients to access their private medical records and to give permission to selected doctors.

#### 3.3.1. Creating Patient Profile

**Figure 20:**
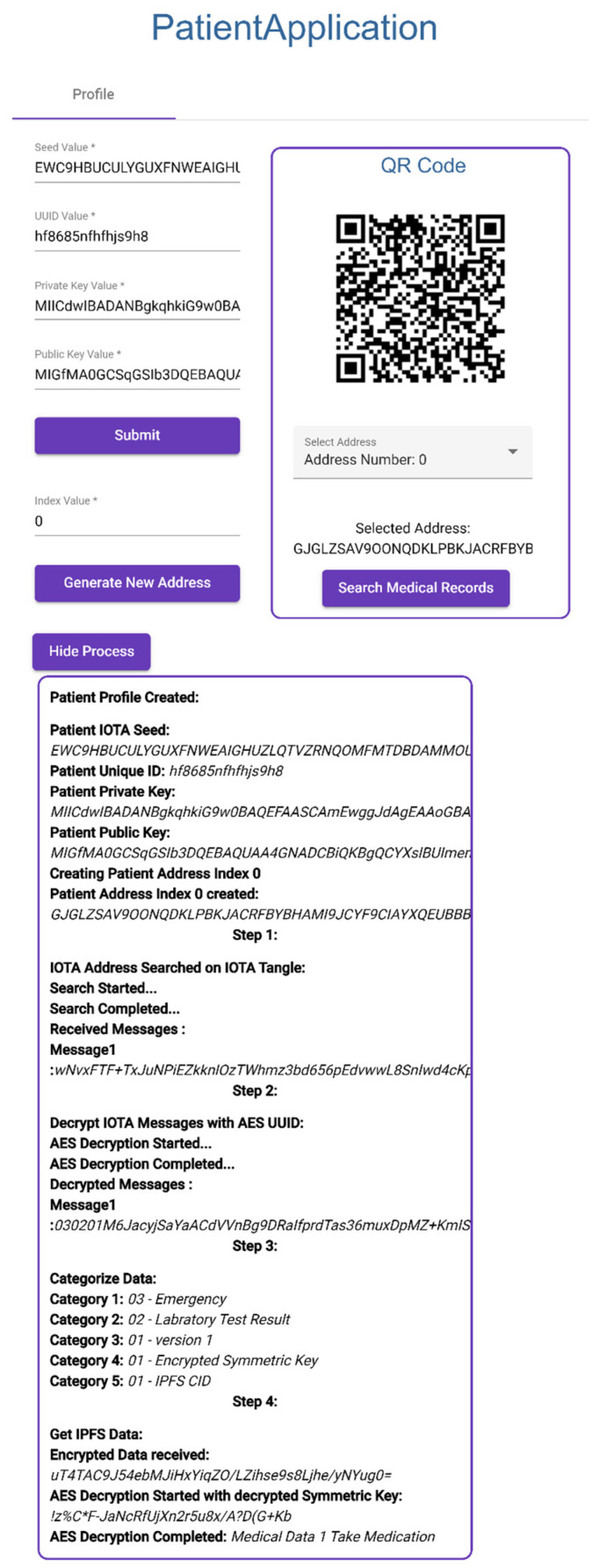
Creating Patient Profile and Searching Medical Records.

- Patient enters IOTA Seed, UUID, Private Key, and Public Key.
- Patient creates new IOTA address with index number.
- Application creates an IOTA address using IOTA Seed and index number.
- Application also creates a barcode to corresponding IOTA address, shown in Figure 20.

#### 3.3.2. Searching Medical Records

- Patient clicks search medical records (Step 1) (Figure 20).
- Patient Application connects to IOTA development server and searches transactions in given IOTA address.
- Messages are extracted from transactions and decrypted with UUID (Step 2).
- Decrypted messages are categorized into five categories (Step 3).
- Medical data are collected from IPFS using IPFS CID (Step 4).
- Symmetric key is decrypted with patient private key.
- Medical data are decrypted with decrypted symmetric key.

#### 3.3.3. Visualizing Medical Records

**Figure 21:**
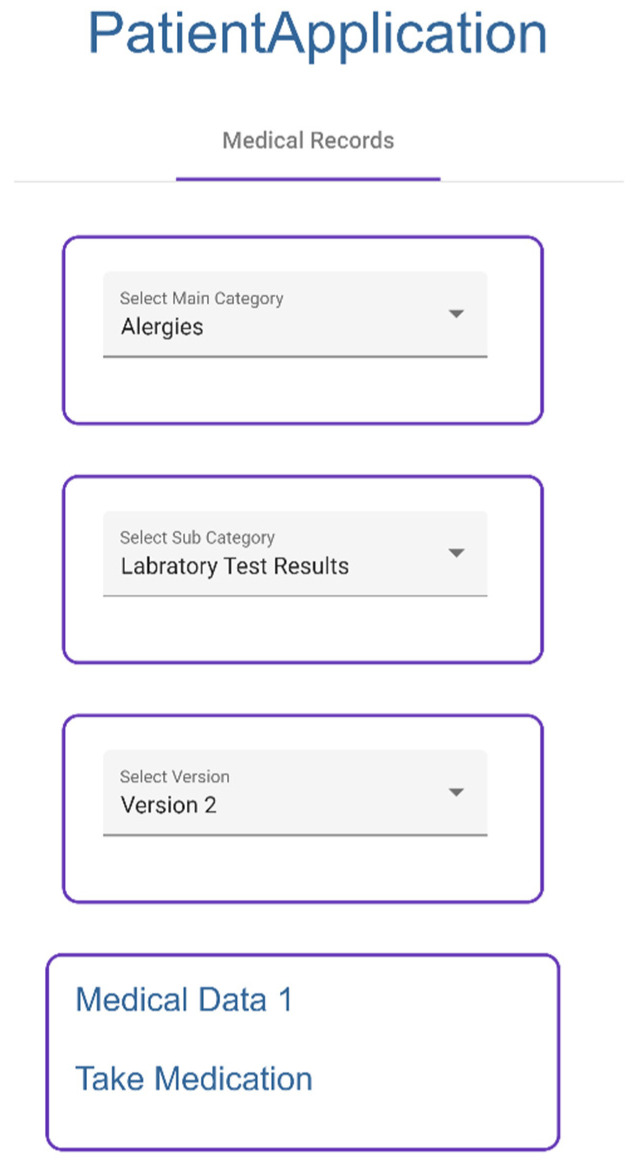
Visualizing Medical Record Patient Application.

- Patient selects given categories to access medical records, which are collected from IPFS (Figure 21).

#### 3.3.4. Giving Permission to Doctor

**Figure 22:**
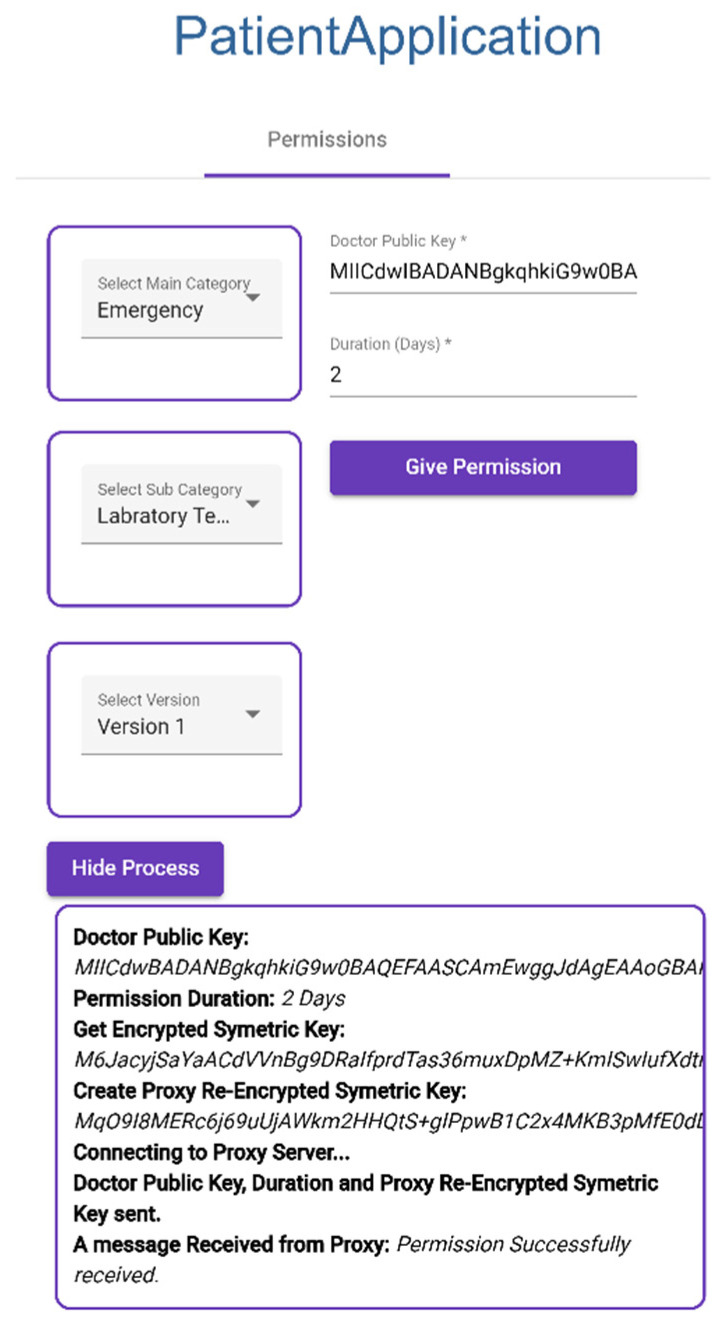
Giving Permission to Doctor.

- Patient selects categories (Figure 22).
- Patient enters doctor public key and duration of permission to selected categories.
- Application collects the symmetric key of the category.
- Application creates re-encrypted symmetric key using doctor public key and symmetric key.
- Application sends doctor public key, duration, and re-encrypted symmetric key to proxy.

### 3.4. Application 4: Remote Medical IoT Device

**Figure 23:**
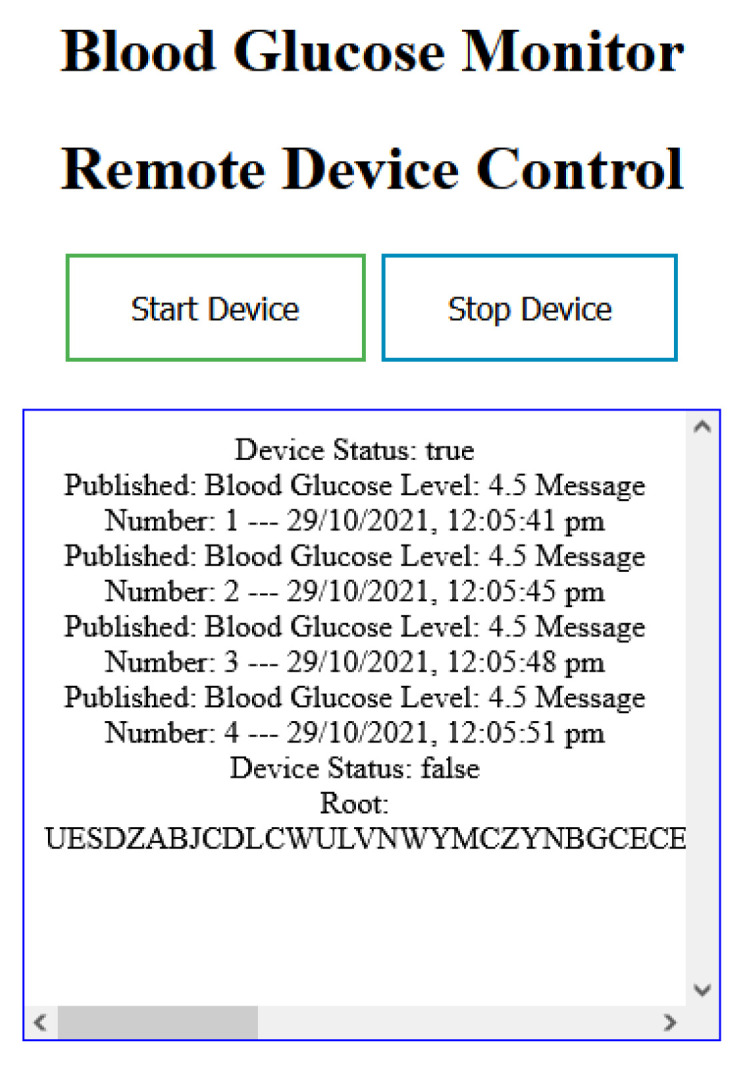
Remote medical IoT device user interface.

This application, which is shown in Figure 23, is developed to simulate remote medical IoT devices. It publishes blood glucose level when the start device is clicked. When the stop device is clicked, it stops publishing data. Published data can be read from a given root address, from the patient application, or from the doctor application.

## 4. Examination and Evaluation of the Proposed Applications

In this section, we present our experimental results and evaluation of the developed application. This assessment is established in terms of scalability, energy efficiency, and decentralisation. Important insights are grounded by analysing the results demonstrating IOTA Tangle’s usefulness for the IOT domain. To do this, we deployed the latest IOTA reference implementation, a Java build personifying the IOTA network specifications on a local server for performing Proof of Work (PoW) operations [61]. The functionality related to IOTA addresses, transactions, routing, and multi-signatures has been implemented using the official Python library of the IOTA Distributed Ledger using iota.lib.py [62]. 

We configured each data node to generate transactions. A set of different Minimum Weight Magnitudes (MWM) (9, 13, 15) is used to identify the effect they have on the Transaction Per Second (TPS) measure. Mainly transactions are broadcast and shared amongst all participant nodes. Two performance metrics are used in this experiment: TPS and Throughput.

**Figure 24:**
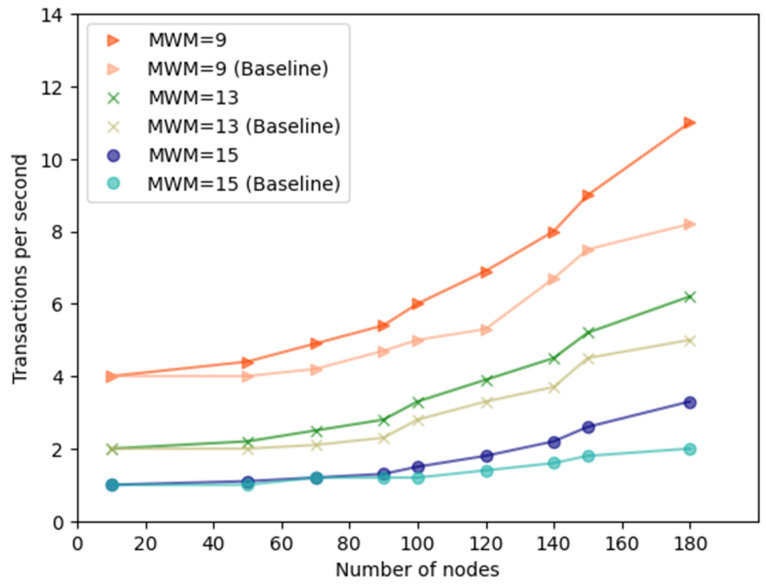
Performance of baseline TPS and developed application under different MWM.

Scalability: As shown in Figure 24, the TPS transaction speed increases linearly when the number of nodes increases. For example, when MWM is 9 and 50 nodes are engaged, the TPS of the application reaches 4.4 tx/s (transaction per second) compared to the baseline TPS, which is 4 tx/s, as shown. Hence, our developed application is 0.4 times faster than the baseline method. When the MWM is 9 and the number of nodes is 180, the TPS reaches 11 tx/s, whereas in the baseline, TPS reaches 8.2 tx/s. This time, the developed application is 2.8 times faster than the baseline method. This validates that our proposed solution is more scalable than the baseline method.

Our proposed solution improves the baseline method in terms of efficiency in processing transactions. For example, when the MWM is set to 15 and 180 modes are engaged, the average TPS of baseline reaches 2 tx/s. When employing our developed application, the average TPS reaches 3.3 tx/s due to computing offloading mechanisms, as presented in Figure 24.

Energy efficiency: The nodes that are performing PoW have an impact on the total energy consumption. The computing offloading preserves energy and reduces the time it takes to process transactions. Our application reduces power due to offloading mechanisms and an allied decrease in the number of transmissions. Figure 24 demonstrates the consequence of MWM on the TPS. In this evaluation, MWM is set to 9, 13, 15 to measure the effect on the TPS. As we can see, the TPS is affected by the use of different MWM configurations; when set to 9, it reaches 11 tx/s, and when set to 15, it reaches 3.3 tx/s.

Decentralisation: Our proposed solution is decentralised, as the consensus mechanism is implemented for usage.

In addition, we measured the performance of traditional computer systems using classical performance metrics: CPU and RAM. We measured the percentage of time our developed application uses the CPU to process the instructions actively. Alternatively, RAM measures the amount of memory used by the developed applications. CPU and RAM are valuable metrics in assessing the overall performance and capacity of the proposed systems.

**Figure 25:**
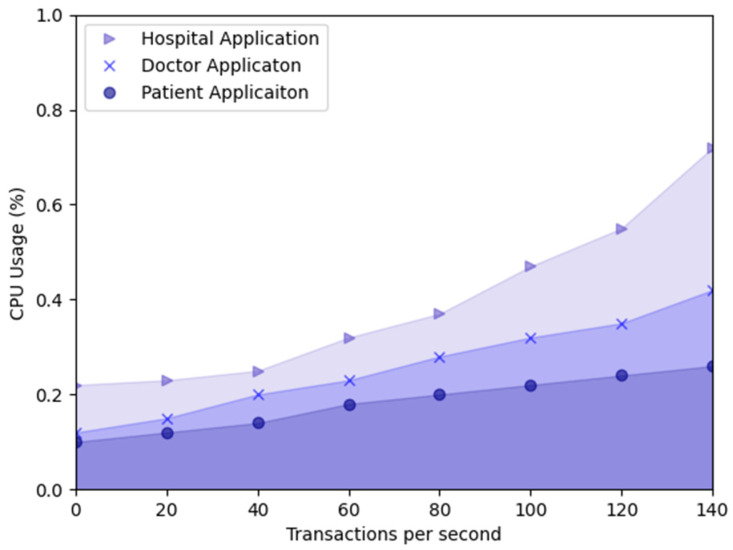
CPU usage of the proposed applications.

Hospital, doctor, and patient applications are developed in this research, and the CPU usage of these applications is measured and presented in Figure 25. As shown in Figure 25, the hospital application uses 0.32% CPU in the TPS of 60 tx/s. Similarly, the hospital application uses 0.55% CPU in the TPS of 120 tx/s. The doctor application’s CPU usage is 0.35% in the TPS of 120 tx/s and the patient application’s CPU usage is 24% in the TPS of 120 tx/s. CPU usage increases TPS transaction speed when the CPU usage increases in hospital, doctor, and patient applications.

**Figure 26:**
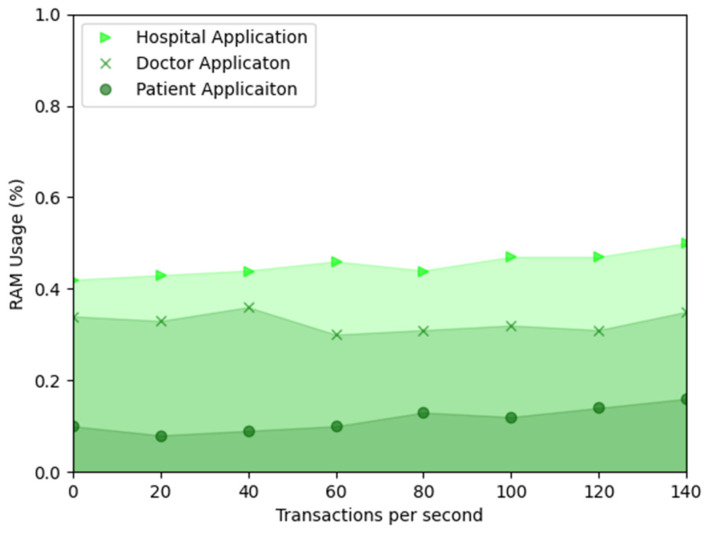
RAM usage of the proposed applications.

The RAM usage of the hospital, doctor, and patient applications are measured and presented in Figure 26. The hospital application uses 0.44% RAM in the TPS of 40 tx/s. Similarly, the hospital application uses 0.47% RAM in the TPS of 120 tx/s. The doctor application’s RAM usage is 0.31% in the TPS of 120 tx/s, and the patient application’s RAM usage is 14% in the TPS of 120 tx/s. RAM usages of the proposed hospital, doctor, and patient applications do not constantly upsurge when transaction speed increases.

Our proposed application of IOTA Distributed Ledger Technology is appropriate to apply to diverse industries that manage personal and sensitive data. Private and secure personal records access management is imperative in various communities and industries. As a distributed ledger technology, IOTA ensures scalability, decentralisation, fast transaction, efficient communication, integration with IOT devices with limited capabilities, and potential for the machine-to-machine economy. By applying this technology, organisations can provide their users with privacy and build trust. The benefit of this research is that the proposed solution can be applied to other industries by modifying the system requirements.

The primary focus of the Internet-of-Things Application is to enable secure and scalable transactions between devices using distributed ledger technology. In this research, IOTA’s DLT can enhance data integrity, privacy, and interoperability in healthcare systems that help secure sharing and access to medical records. The benefit of IOTA’s technology is its broader applicability across various industries. This technology allows devices to securely communicate and share data in a decentralised and scalable manner and enables machine-to-machine (M2M) data integrity and trustless interactions between IoT devices. Moreover, IOTA can be applied to energy systems to facilitate the decentralisation of energy trading and management, allowing peer-to-peer energy transaction and grid optimisation and facilitating the integration of renewable energy sources. In addition, IOTA’s DLT can be used to track and trace goods throughout the supply chain, ensuring transparency and immutability of data. This can also enhance the efficiency of supply chain processes, inventory management improvement, fraud reduction, and automated and secure transaction between stakeholders. IOTA technology can provide a secure data exchange from various city systems, such as energy, waste management, and transportation, contributing to building more innovative and efficient communities.

## 5. Discussion

In the framework, PHR is stored in IPFS. The IOTA Protocol is used to store IPFS hashes, to generate smart contracts, and to communicate securely with IoT devices using IOTA MAM. To achieve decentralization, an IOTA distributed ledger and IPFS protocols are used. What makes IOTA preferable compared to the other distributed ledger technologies is the Tangle technology. Tangle technology overcomes two fundamental disadvantages of blockchain: transaction costs and scalability. 

This framework will also bring clarity to hospital expenses in terms of patient care fees. In Australia, hospitals are calculating the cost by Activity-based funding (ABF), which is the number of services provided to patients [63]. In the proposed framework, every medical IoT device that is used for medication is registered in smart contracts, and payments are collected over time based on consumption.

Energy consumption is another issue that IOTA Tangle technology tries to solve by eliminating miners. There are no blocks compared to blockchain, thus allowing IoT Tangle to be scalable. For instance, the estimated power consumption of Bitcoin is 0.1 to 10 GW due to PoW calculations for blocks and cooling the machines [64]. In IOTA, power consumption is reduced with periodical snapshots by resetting transaction history. Thus, compared to other blockchain-based systems proposed in the literature, the computing cost is expected to be reduced in the proposed framework.

One of the main advantages of IOTA is the lack of miners; therefore, there is no fee for transactions, making it suitable for IoT devices in terms of machine-to-machine transactions. However, during periodic snapshots taken by the IOTA Foundation, zero value transactions messages and zero value addresses are removed from Tangle to make it lightweight, increase transaction speed, and decrease the power consumption of nodes. Unless it is recorded in Permanodes or histories of transactions are manually extracted, the deleted history of transactions cannot be restored. This poses a problem in the framework for medical headers, which is saved in transaction messages linked to the Patient IOTA Address. To save prior medical headers, an API that automatically collects past transactions of Patient IOTA Address must be established. This API must automatically store previous transactions in IPFS and retrieve them after the snapshot is completed. Snapshots are also a challenge in terms of service availability. However, IOTA development is in the beta stage, and the IOTA Foundation has announced that in the future, snapshots will be automated for each node.

Limitations of the framework include the fact that IOTA is not completely decentralized because of coordinator nodes. To confirm transactions, the current IOTA protocol still relies on coordinator nodes, which are administered by the IOTA Foundation. As a result, it cannot be called a completely decentralized distributed ledger. However, IOTA is in the development phase, and the IOTA foundation recently introduced Coordicide to eliminate coordinator nodes [52] to make it completely decentralized. Another concern is that even though IOTA transactions are free, smart contracts, on the other hand, require a fee for computational effort. However, the IOTA Smart Contract Protocol allows users to choose their own committee of nodes; thus, hospitals can use their own computer to reduce the cost of smart contracts.

In the initial development, Proxy Re-encryption was considered to be used for all data that are stored in IPFS. In this way, the patient could have encrypted all data with a public key without revealing the private key to the doctor. However, Proxy Re-encryption is very slow for big data [43]; therefore, Proxy Re-encryption is only used for encryption of 256-bit symmetric key, which is shared with doctors to decrypt IPFS data.

Another limitation is that distributed storages, such as IPFS, which is used in the framework, is not acceptable in certain countries, such as the USA and Australia, according to their legislation, including HIPAA [65] and the My Health Records Act, where patient data storage location is unknown. These laws were set with the concern of availability and accessibility of the data. However, this can be solved by creating a private IPFS network or assigning servers that are always connected to the IPFS network in hospitals. However, this will oppose the purpose of using the IPFS distributed ledger for decentralizing patient records and reducing hospital expenses.

## 6. Conclusions

Traditional patient electronic health record systems are expensive, complex, centralized, and often insecurely store patient data. Furthermore, patient confidentiality and privacy are not prioritized in many systems. This study focused on designing a fully decentralized PHR using IOTA Tangle to secure patient medical records and IOT medical devices and to create an access management system that gives patients full control of their own medical records. 

The proposed framework can improve healthcare services by providing immutable, secure, scalable, trusted, self-managed, and traceable patient health records. IOTA technology eliminates miners, and it enables feeless micro transactions, secure communication between IOT devices, and low-cost smart contracts, which are fundamental components of the framework.

In the next phase, a prototype system will be developed to simulate the behavior of the framework. The first step of the work is developing a Web application that will extract IOTA transactions from the given IOT address. The second step is developing a mobile application that will be used by patients to give access to authorized people by using smart contracts. The third and last step is developing a web application that will be used by a doctor for Proxy Re-encryption for key exchange with patients.

## Figures and Tables

**Table 1:** Comparative analysis between the existing solutions.

Existing Solutions											
Key Contexts	Rezaeibagha et al. [17]	Semantha et al. [13,28]	OmniPHR [26]	Healthchain [29]	Thwin and Vasupongayya [30]	Meier et al. [31]	Hussien et al. [32]	Skaly [35]	Smart Optz [36]	Pact [37]	Our Proposed Framework
IOTA Tangle [38]								⚫	⚫	⚫	⚫
Distributed Ledger Technology (DLT) [39]		⚫	⚫	⚫	⚫		⚫	⚫	⚫	⚫	⚫
IPFS protocols [40,41]		⚫					⚫				⚫
Application Programming Interface (API) [42]										⚫	⚫
Proxy Re-encryption (PRE) [43,44]					⚫						⚫
Access control [30]	⚫	⚫			⚫	⚫	⚫				⚫

**Table 2:** Hospital admission flowchart steps.

Step	Description
Step 1	Patient uses Hospital Web Application to make an appointment. Patient shares IOTA address and UUID.
Step 2	Patient obtains Doctor Public Key from Hospital Web Application. Patient uses Patient Application to give permission to selected Patient Medical Records using Doctor Public Key.
Step 3	Patient Application creates Re-encryption Key using Patient Private Key and Doctor Public Key. Patient Application uses Re-encryption Key to re-encrypt symmetric key that belongs to permissioned Medical Record.
Step 4	Patient Application creates parameters of the Smart Contract 1 (Doctor Public Key, Permissioned Medical Header, duration of permission, and re-encrypted symmetric key).
Step 5	Patient Application creates Smart Contract 1 to share proxy location with doctor for a limited period of time.
Step 6	Smart Contract shares Doctor Public Key, Permissioned Medical Header, duration of permission, and re-encrypted symmetric key with a proxy.
Step 7	Hospital receives patient information from Hospital Web Appointment and creates Smart Contract 2 to validate patient and patient’s insurance. Then, fixed amount of IOTA tokens from insurance or patient IOTA address is withdrawn.
Step 8	Doctor searches patient appointment from Doctor Application (PDV). This information is received from Hospital Web Appointment Application.
Step 9	PDV connects to IOTA node and searches patient address in IOTA Tangle.
Step 10	PDV uses patient address to find old medical transactions in IOTA Tangle.
Step 11	UUID encrypted transaction messages are extracted from messages.
Step 12	Transaction messages are decrypted with UUID symmetric key.
Step 13	Doctor searches patient smart contract. Smart contract validates doctor and sends proxy location.
Step 14	Proxy validates Doctor using signature algorithm and sends medical header and corresponding re-encrypted symmetric key.
Step 15	PDV uses Doctor Private Key to decrypt re-encrypted symmetric key.Patient records are downloaded from IPFS using IPFS hash and decrypted with decrypted symmetric keys and stored in PVR temporarily.
Step 16	After patient records are visualized by PDV, doctor assigns IOT devices to patient using PDV and creates smart contracts with each assigned IOT Device.
Step 17	Each IOT device has a separate smart contract that is linked with Smart Contract 2 to request balance during medication.
Step 18	Smart Contract 3 updates Account Balance during medications.
Step 19	If needed, more IOTA tokens are taken from Smart Contract 2.

**Table 3:** Patient discharge flowchart steps.

Step	Description
Step 21	Doctor creates new patient medical data from Doctor Application (PDV).
Step 22	PDV (Patient Data Visualizer) categorizes data according to Medical ID, such as test results, treatments, etc..
Step 23	After data are collected over a period of time, all data encrypted with random AES-256 symmetric key.
Step 24	Encrypted data uploaded to IPFS.
Step 25	IPFS hash address created.
Step 26	Symmetric key encrypted with patient public key.
Step 27	Using Encrypted symmetric key, IPFS Hash, and Medical ID, a medical header is created.
Step 28	Medical header encrypted with patient UUID and saved in IOT transaction message.
Step 29	Hospital sends patient discharge information to Smart Contract.
Step 30	Smart Contract validates insurance and sends unused IOTA tokens to patient IOTA address.

**Table 4:** Remote patient health record flowchart steps.

Step	Description
Step 31	Patient accesses the IOT device and starts IoT device.
Step 32	Patient logs into device interface.
Step 33	Patient obtains IOTA MAM Root Address and side key.
Step 34	Patient registers new IOT device using IOTA MAM Root Address.
Step 35	Patient application collects data from root address.
Step 36	After data are collected over a period of time, all data are encrypted with random AES-256 symmetric key.
Step 37	Encrypted data are uploaded to IPFS.
Step 38	IPFS hash address is created.
Step 39	Symmetric key is encrypted with patient private key.
Step 40	Using Encrypted symmetric key, IPFS Hash, and Medical ID, a medical header is created.
Step 41	Medical header is encrypted with patient UUID and saved in IOT transaction message.

## Data Availability

Not applicable.

## Abbreviations

ABF	Activity-based funding AES
AFGH	Ateniese, Fu, Green, and Hohenberger
API	Application Programming Interface
CID	Content Identifier
COVID-19	Coronavirus Disease 2019
DAG	Directed Acyclic Graph
DLT	Distributed Ledger Technology
EHR	Electronic Health Records
GW	Gigawatt
HIPAA	Health Insurance Portability and Accountability Act
HL7	Health Level Seven International
FHIR	Fast Healthcare Interoperability Resources
ID	Identity
IoT	Internet of Things
IoMT	Internet of Medical Things
IPFS	InterPlanetary File System
IPLD	InterPlanetary Linked
MCMC	Markov chain Monte Carlo
MHR	My Health Record
MAM	Masked Authenticated Messaging
PDV	Patient Data Visualizer
PHR	Personal Health Records
PRE	Proxy Re-encryption
PoW	Proof of Work
P2P	Peer-to-Peer
SHA	Secure Hash Algorithm
QR	Quick Response
URL	Uniform Resource Locator
USA	United States of America
UUID	Universally Unique Identifier
